# Optimising Outcome in Congenital Hypothyroidism; Current Opinions on Best Practice in Initial Assessment and Subsequent Management

**DOI:** 10.4274/Jcrpe.849

**Published:** 2013-03-01

**Authors:** Malcolm Donaldson, Jeremy Jones

**Affiliations:** 1 Glasgow University, Royal Hospital for Sick Children, Child Health Unit, Glasgow, United Kingdom

**Keywords:** congenital hypothyroidism, thyroxine, thyroid ultrasound, radioisotope scanning

## Abstract

Congenital hypothyroidism (CH), usually of the primary and permanent variety, is an eminently preventable cause of growth retardation and mental handicap whose outlook has been transformed by newborn screening, usually involving the measurement of capillary thyroid stimulating hormone (TSH). Severe primary CH, due for example to athyreosis, may result in subtle cognitive, behavioural and sensori-motor deficits, but the extent to which these can be offset by optimal postnatal diagnosis and management remains uncertain. This is because the available adult follow-up data reflect the outcome of previous management in the 1970’s and 1980’s, and also because the accurate neuro-psychological assessment of children is difficult, particularly in the preschool population. There is an urgent need to develop new consensus guidelines and to ensure that the children managed according to such guidelines are systematically and prospectively assessed so that good quality outcome data become available. In this review, key recommendations in the management of CH include: screening at day 3 so that severely affected infants can begin treatment within the first 10 days of life; setting the TSH referral cut-off at 8-10 mU/L; adopting a disciplined diagnostic algorithm to evaluate referred cases, with measurement of venous free thyroxine (T4), TSH and thyroglobulin combined with dual ultrasound and radioisotope imaging; initial treatment with a T4 dose of 50 μg daily in infants weighing ≥ 2.5 kg and 15 μg/kg/day in infants weighing < 2.5 kg followed by weekly review until thyroid function is normalised; and maintenance of free T4 levels between 15-26 pmol/L and TSH between 0.5-5 mU/L thereafter to avoid both under- and overtreatment.

**Conflict of interest:**None declared.

## INTRODUCTION

Newborn screening for congenital hypothyroidism (CH) was pioneered in the late 1970s and established in most Western European countries, North America and Australasia by the mid-1980’s. In countries that can afford newborn screening, treatment within the first 28 days of life - so-called ‘early treatment’ - has transformed the outlook for children with CH so that severe growth retardation with mental handicap (cretinism) is no longer seen. Although the prevalence of frank learning disability intelligence quotient (IQ <70) in children diagnosed clinically, prior to the introduction of newborn screening, is lower than previously thought at 8-25% (1), the case for newborn screening to prevent mental handicap is overwhelming. Indeed, CH is one of the few conditions in which the cost of newborn screening is less than the cost of managing the sequelae of late diagnosis (2). 

Although the outcome in infants detected through newborn screening is greatly superior to that of infants who are only detected after clinical signs have developed, there is nevertheless evidence for neurological deficit in some of this early-treated population. The purpose of this review is to first appraise the evidence for neurological impairment in early-treated subjects with CH and to consider which factors may influence outcome. We then focus on current opinion as to best practice in the detection, early assessment, and management of infants who are referred with raised thyroid stimulating hormone (TSH) on newborn screening. Our recommendations are drawn with reference to current UK and Scottish guidelines; from our personal experience at the Royal Hospital for Sick Children in Glasgow; and from the European Society for Paediatric Endocrinology (ESPE) International Consensus Meeting on Congenital Hypothyroidism which was held in Rome in November 2011 and at which one of us (MD) was a participant. 

**Definitions and Prevalence**

According to the United States Rare Diseases Act of 2002, CH falls into the category of a rare disease (prevalence less than 1 in 1500) but is nevertheless the commonest paediatric endocrine disorder. It is described as primary when the gland itself is affected and central when the defect lies in the hypothalamo-pituitary axis; compensated when the hypothalamo-pituitary-thyroid axis is jeopardised but still manages to maintain normal thyroxine (T4) levels and decompensated when normal thyroid hormone levels cannot be maintained. The childhood and adult reference ranges for total and free T4 (fT4) are 80-160 nmol/L and 9-26 pmol/L, but during the first two weeks of life, T4 levels may be as high as 68 pmol/L, falling to 12-30 pmol/L thereafter (3). 

There are no standard definitions concerning the severity of CH, but this can be stated in relation to the pre-treatment venous fT4 level as: severe = fT4 <5 pmol/L; moderate = 5-≤ 10 pmol/L; and mild =10-15 pmol/L. Primary CH is usually permanent but may be transient, especially in sick and preterm infants. Its prevalence amongst unscreened populations is about 1 in 6000 births (1), increasing to 1 in 3000-4000 when newborn screening is performed with Scottish data showing 1 in 4363 affected infants in 1979 -1993, increasing to 1 in 3655 in 1994-2003 (4). The considerable variation in prevalence for primary CH relates partly to ethnic differences but also reflects iodine status, the TSH cut-off levels used for referral, and the rigour with which true permanent CH is distinguished from transient TSH elevation (5). The purely descriptive term hyperthyrotropinaemia refers to mild TSH elevation with normal T4 levels of uncertain cause. Neonatal hypothyroxinaemia, defined as low T4 levels in the context of normal TSH, is a distinct but important phenomenon which is almost exclusively seen in sick and/or preterm infants, and which appears to be an independent predictor for neurodevelopmental problems (6). 

Central CH is rare, although recent estimates of around 1 in 20 000 births are higher than previously reported (7). Central CH is almost always associated with other anterior pituitary hormone deficiencies. 

**Causes of Congenital Hypothyroidism**

The causes of CH are shown in Table 1. In almost all cases, the thyroid problem is isolated, but syndromic forms, particularly those related to gene defects in the transcription factors TTF-1, TTF-2 and PAX8, may be seen in the context of other congenital malformations (8). 

Most primary CH is due either to abnormal development of the gland itself-dysgenesis; or to deficiency in one of the enzymes of thyroid biosynthesis within a structurally normal thyroid gland-dyshormonogenesis. Thyroid dysgenesis is the commonest cause and is mostly due to ectopia (usually sublingual). True thyroid hypoplasia is rare and its presence should prompt investigation for a mutation in the TSH receptor gene or in PAX8 (9,10) When the gland cannot be seen on thyroid imaging, the term athyreosis is preferable to the more presumptive ‘thyroid agenesis’ and can be usefully sub-classified into apparent athyreosis when thyroglobulin (TG) is detectable, and true athyreosis when it is not (11). 

Apparent athyreosis may be due to an ectopic gland that is too small and poorly functioning to be seen on thyroid imaging and also to inactivating TSH receptor mutations (12). Transient TSH elevation, with or without low T4 levels, is typically seen in sick infants (e.g. in association with prematurity, respiratory distress, hypoxia, and congenital malformations) (5) but is also seen in states of iodine deficiency and excess, and due to transplacental transfer of blocking thyroid autoantibodies. Spurious capillary TSH elevation results from assay problems, and in some cases, no cause is found. 

**Outcome in Early-treated Congenital Hypothyroidism**

The surprisingly good prognosis in severe primary CH can be explained by transplacental supply of maternal T4 (13). Provided that maternal thyroid hormone and iodine status are normal, enhanced conversion to the active thyroid hormone triiodothyronine (T3) in the fetal cerebral cortex due to increased activity of type II deiodinase (D2) (14) will protect the fetal brain from severe neurological impairment. However, impaired maternal thyroid status, due for example to iodine deficiency or autoimmune hypothyroidism, has an adverse effect on fetal brain development (15,^16^), while the rare combination of maternal and fetal hypothyroidism will lead to more severe neurodisability. 

The problems reported in early-treated subjects with CH can be broadly grouped into two categories as cognitive and behavioural, including deficits in IQ, memory and attention; and sensori-motor, including problems with fine motor, balance and auditory function. 

**Cognitive and Behaviour Problems**

The prevalence of frank learning disability in screened populations with CH is very low. Only 1.4% of Scottish patients diagnosed between 1979-1993 attended special schools (17), while Rovet and Ehrlich found that 4% of Canadian patients were in special education at Grade 6 level compared with 7% of controls (18). 

There are however data to show an IQ deficit in some children with early-treated CH. Tillotson et al (19) reported a 10.3 point IQ deficit in severe (initial T4 <24.8 nmol/L) CH at age 5 years; Rovet et al (20) noted an 8 IQ point difference between 42 children with CH and their unaffected siblings at 6 years; and Salerno et al (21) found a subnormal IQ in 13 of 40 patients with CH at 12 years. In adults aged 21 years, Kempers at al (22) showed a mean full-scale IQ of 91.3 in 35 patients with severe hypothyroidism (T4 <30 nmol/L at diagnosis) compared with 101.3 in 19 mildly affected patients (T4 ≥60 nmol/L). Concerning memory, behaviour and problems related to attention, Bongers-Schokking et al (23) 2005 study reported more problems with aggression and attention in the patient group compared with sibling controls. The work of Rovet and colleagues confirms that some children with CH have difficulty with concentration (18). In adulthood, Oerbeck and colleagues from Norway found that 49 adults (mean age 20 years) with CH scored significantly lower than their 41 sibling controls in tests of memory (especially story recall) distractibility, and also self-reported more behaviour problems (24). However, this group was unable to confirm the link between high T4 dosage in infancy and behaviour problems in later childhood reported by Rovet and Ehrlich in 2000 (18). 

**Sensori-motor Problems**

Earlier studies during childhood from the Netherlands and Toronto particularly have reported visual-motor impairment during childhood in CH (23,25,26,27). In the UK, Simons et al (28) reported decreased motor skills at 10 years of age in 59 children with CH born between 1978-81 compared with controls, the difference being more marked in the 31 cases with pretreatment T4 levels of ≤40 nmolL. Fewer data are available in adulthood, but Kempers et al (22) study confirmed reduced manual dexterity and ball skills in the 35 severely affected patients. 

Hearing impairment in CH is of considerable interest. David Grant’s group in the UK reported mild hearing impairment in childhood (29), and abnormal auditory brainstem evoked potential tracings were found in 7 out of 7 patients with CH, 6 of whom had low T4 levels (30). In young adults, work from Oerbeck et al (31) has suggested problems with auditory processing and selective attention. The recent questionnaire-based report from France by Léger and colleagues in 1202 patients diagnosed between 1978 and 1988 has shown a surprisingly high prevalence of hearing impairment compared with controls (9.5% versus 2.5%), highlighting the need for monitoring throughout childhood and beyond (32). 

**Interpretation of the Available Outcome Data in Congenital Hypothyroidism**

There is overwhelming evidence that the severity of CH is a major factor predicting outcome, patients with athyreosis faring worse than those with other forms of thyroid dysgenesis such as sublingual ectopia (25,26,27,28). Previous work claiming that any IQ deficit could be reversed by early, high-dose, T4 treatment was, in retrospect, over-optimistic being based on small numbers of patients aged only 18 months (33). However, postnatal management and compliance with treatment have been shown to affect outcome, a French cohort study of 682 patients born between 1979 and 1985 showing that inadequate treatment in childhood was an independent factor in school delay (34). 

At present, the extent to which optimal postnatal treatment can offset intrauterine deficit from severe CH remains uncertain, and it is important to judge the information from existing studies in the context of the subjects concerned usually being born before or during the early 1980’s. The median age at treatment, for example 28 days in the cohorts of Salerno et al (21) and Kempers et al (22) and 17 days in that of Tillotson et al (19), is later than the current target of < 13 days (35), while most subjects received lower doses of T4 - 8-10 μg/kg/day compared with the higher doses of 10-15 μg/kg/day which are now recommended (36). Data on more recently treated patients is more favourable - 45 children with CH born between 1993 and 1996 in the Netherlands and tested at 5-7 years showed similar mean global IQ scores compared with 37 controls (104.7 vs. 105) although children with severe disease (athyreosis or total dyshormonogenesis) had lower visuo-motor scores than the more mildly affected patients (23). 

**Screening, Diagnosis, Assessment, and Management of Congenital Hypothyroidism with Reference to European, UK and Scottish Guidelines**

**Capillary Screening Methods and Hormone Cut-Offs**

The technique of capillary screening involves sampling from a heel-prick stab within a few days of birth and drying the blood onto circles on prepared filter paper which is then mailed to a regional laboratory. Assays are available for the measurement of total T4 (it being impractical to measure fT4 on blood spot samples), TSH and thyroid binding globulin (TBG). 

**Figure 1:**
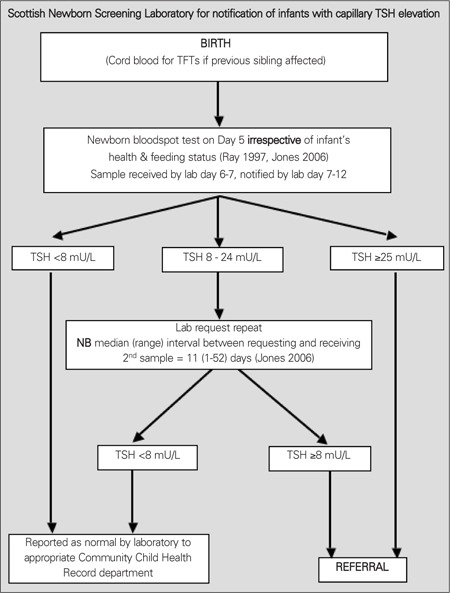
Protocol for laboratory notification of babies with TSH elevationdetected on newborn screening in Scotland

Despite a few notable exceptions in the USA and the Netherlands, most screening programmes measure TSH alone. Figure 1 shows the current protocol for Scotland where blood spot TSH values of <8 mU/L are reported as normal, values ≥25 mU/L prompt immediate referral to a clinician, while values of 8-25 mU/L result in a repeat sample being requested, with referral if the repeat capillary blood spot TSH is ≥8 mU/L (equivalent to a serum value around 16 mU/L). 

An alternative is a two-tiered technique in which total T4 is tested initially, followed by TSH measurement when T4 is below an agreed cut-off, for example - 0.8 standard deviation (SD) or less than the mean score for the day of testing. This technique has the advantage of detecting central as well as primary hypothyroidism, but its sensitivity in diagnosing the former is limited (37) since total T4 is influenced by other factors such as protein binding. Colleagues from the Netherlands have described an elegant refinement in which the sensitivity of T4 measurement is enhanced by also measuring TBG when T4 is-1.6 SD or less, and hence deriving the T4:TBG ratio (a surrogate of fT4) (38). During a 2-year prospective study, this group found a prevalence for central CH of 1 in 16 404 children and estimated that 5% or less of primary and central hypothyroidism would be missed using this technique, as opposed to 15-20% using either TSH alone or the two-tiered T4 and TSH system (39). The extra cost of the T4/TBG addition was estimated at $11 206 per additional case detected. Despite favourable reports of the three-tier system employed in the Netherlands, most European countries use the TSH only method, recognizing that most (although not all) cases of central hypothyroidism will be detected clinically in the context of other anterior pituitary abnormalities. 

If TSH screening alone is used, what should the cut-off be? In Greece, reducing the TSH cut-off from 20 mU/L to 10 mU/L led to the discovery of CH in 56 of 200 patients diagnosed between 2000 and 2002, with a 10-fold increase in recall rate (40). Cheetham’s group in Newcastle have shown that even a blood spot cut-off of 10 mU/L (equivalent to a serum value around 20 mU/L) will miss mild cases of CH and have suggested that the increased detection with a level of 6 mU/L may justify the extra cost and higher recall rate (41). However, Krude and Blankenstein have questioned the evidence for the benefit of detecting TSH level between 6-10 mU/L and highlighted the potential harm of false-positive results in terms of causing parental distress (42). 

**Optimal Timing of Capillary Blood Spot Sampling and Notification**

In high risk cases (e.g. when a sibling is affected), cord blood should be sampled for TSH, but as a rule, screening within the first 48 hours of birth should be avoided since the TSH surge occurs during this time (43). The recommended age at screening in the UK is day 5 (44). In Scotland, the median age at screening of referred infants from 1994-2003 was 6 days (recommended age 4-6 days), with notification and treatment at 10 and 11 days (4), but a recent European survey indicated that the median age of screening in most countries is now 3 days (Gerard Loeber, personal communication). 

While delay in starting treatment beyond one month of age in severe CH is known to adversely affect IQ, the benefit of starting treatment particularly early (e.g. < 15 days) has been surprisingly difficult to prove. Boileau et al (45) found IQ outcome to be related to age at starting treatment in 131 children recalled at mean (range) 22.8 (3-85) days, with an apparent threshold at 21 days. However, the children treated < 21 days had also received a larger dose of T4 (5.8 vs. 5.3 ug/kg/day). The outstanding study of Leger et al (32) on 1201/1748 patients diagnosed during the first decade of screening in France and treated at a median (25^th^-75^th^ centile) age of 20 days (16-25 days) did not show age at starting treatment to be a factor in educational attainment. Similarly, Kempers et al (22) were unable to find a correlation between age at starting treatment and cognitive and motor outcome in their 70 adult patients who had started treatment at a mean age of 28 days. Bongers Schokking et al (46) reported improved IQ in 61 children with CH tested at 10-30 months of age who had started treatment before 13 days compared with ≥13 days but could not confirm this on subsequent testing on 45 of the original cohort at 5-7 years (23). Part of the reason for the difficulty in demonstrating a correlation between age at starting treatment and outcome is that, within any cohort studied, the variation in the age at starting treatment may not be sufficiently wide to show differences that do in fact exist. 

Moreover, there are commonsense grounds for starting T4 treatment (and hence undertaking newborn screening) as soon after birth as possible. It is known that intrauterine hypothyroidism in babies with athyreosis adversely affects outcome and that diagnosis beyond one month leads to neurological impairment. It is logical, therefore, to minimise the gap between the abrupt discontinuation of maternal T4 supply at birth and the initiation of postnatal treatment in order to protect infants with severe disease. Moreover, severe hypothyroidism is known to cause unwanted symptoms during the postnatal period including feeding difficulties, prolonged jaundice and hypothermia, all of which the clinician wishes to avoid. There is, therefore, a case for carrying out newborn screening for CH on day 3 of life, once the logistics of screening for other disorders in the newborn period have been taken into consideration, so that treatment can be started within the first 8-10 days of life. Discussions are underway in the UK for this change to be implemented. 

**Figure 2:**
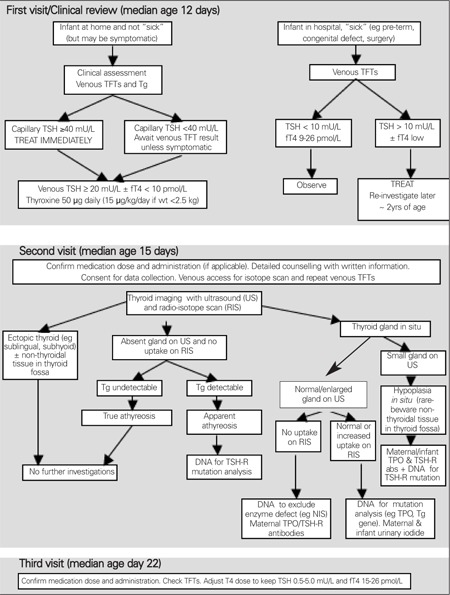
Flow chart for the diagnosis and management of infants referredwith capillary TSH elevation (≥25 mU/L initially or ≥8 mU/L on repeat) in theWest of Scotland

**Initial Visit:** Clinical assessment, venous sampling, counselling, and initial treatment Figure 2  shows the diagnostic and management sequence that we adopt in the West of Scotland for infants referred with TSH elevation on newborn screening. 

**Clinical Assessment**

Initial clinical assessment following notification should include a thorough history and examination as shown in Table 2.

Cardiac and hip examination should receive special attention given the reported increase in congenital heart disease [14/243 (5.8%)] – and developmental dysplasia of the hip [8/243 (3.8%)] in one series (47), although the Scottish prevalence figures from 1979 – 2010 are lower at 8/525 (1.5%) and 6/525 (1.1%) (Jones and Donaldson, in preparation). Dysmorphic features should be sought since syndromic disorders may be associated with either transient TSH elevation (5) or permanent hypothyroidism due to TSH resistance in the case of Albright’s hereditary osteodystrophy with mutation in the GNAS gene. 

**Venous Sampling**

Because of the diagnostic, therapeutic and prognostic importance of the initial, pre-treatment thyroid hormone level, a good venous sample (at least 1 mL) of heparinised blood should be taken prior to treatment together with 1 mL of clotted blood for TG measurement. 

At the initial visit, additional blood may be taken for thyroid peroxidase and TSH-receptor antibodies from both mother and infant if there is a family history of thyroid disease, and DNA samples from both parents and infant with a family history of consanguinity. Otherwise, these investigations may be deferred until thyroid imaging has been carried out (see Figure 2). 

**X-ray of Knee**

This is practised in some centres since bone retardation on knee X-ray reflects the severity of intrauterine hypothyroidism and has been shown to correlate with IQ outcome (48).

**Initial Counselling**

At the first visit, the parents are often deeply shocked by the disclosure that their apparently perfect baby may have a lifelong thyroid disorder. It should also be remembered that the mother will usually be less than 2 weeks post partum and may be experiencing some of the common difficulties with sleep pattern and feeding that occur at this time. In our view, extensive counselling and explanation, and thyroid imaging, should not be carried out at this stage. Instead, we advise simply informing the family that the baby probably does have an underactive thyroid gland which will require lifelong treatment and that the outlook is excellent provided that the T4 replacement treatment is given faithfully, and ensuring that the family understand what medication to give, and how. 

**Initial Treatment**

Unless capillary TSH elevation is relatively mild (below 40 mU/L), we advise starting T4 treatment without waiting for the venous results. Given the evidence for better IQ outcome using higher, rather than lower doses of T4, we advocate an initial T4 dose of 50 μg daily in infants weighing ≥2.5 kg and 15 μg/kg/day in infants weighing <2.5 kg, consistent with previous European guidelines (49).

There are few published data on the mode of T4 administration. A survey we conducted amongst British paediatricians showed that the majority recommended giving T4 in tablet form, crushed in 5 mL of milk or boiled water, either using teaspoon or syringe (50). Since then, the liquid T4 solution, Evotrox (Orbis Consumer Products Ltd, Middlesex, UK), has become more widely used in the UK (Dr Catherine Peters from GOS, personal communication) although there are at present no data on its use in infants or children. Until such published data comparing the reliability and effectiveness of liquid solution versus tablet T4 is available, we continue to recommend T4 in tablet form, crushed and given in a syringe with 2-5 mL of boiled water or milk. 

If Evotrox is used, great care should be taken to ensure that the correct strength (25 or 50μg/5mL) is consistently prescribed so as to avoid confusion and consequent over- or under-dosage with T4. 

Whatever T4 preparation is used, the dispensing pharmacy must ensure that the parents fully understand how to prepare and give the medication. Parents should be encouraged to err on the side of repeating the medication in the event of minor vomiting after giving medication. There is a strong case for providing written instructions regarding T4 administration, something that is not currently advocated by UK or ESPE guidelines. 

Details on procedure (diagnostic imaging, further investigation, and definitive counselling) to be followed at the second visit are given in Figure 2.

**Diagnostic Imaging**

We advise carrying out both thyroid ultrasound and isotope imaging in all babies referred with TSH elevation provided that they are fit for transfer to a specialist centre, in contrast to preterm and sick babies who are unable to leave the neonatal unit. T4 treatment will affect isotope uptake if TSH is suppressed to <5 mU/L, but this is unlikely if isotope scanning is carried out within 5 days of starting T4 treatment and, as indicated above, we recommend carrying out scanning at the second visit, 3-5 days after starting treatment. Whatever decision is taken, as to the timing of thyroid imaging, this should never be allowed to postpone T4 treatment. 

We recognise that not all centres, especially in the UK, either carry out or advocate diagnostic thyroid imaging in infants referred with TSH elevation. However, we strongly recommend this approach since:

• Imaging aids genetic counselling. In the case of dyshormonogenesis, it ensures the appropriate targeting of further molecular genetic study and also early diagnosis (with a cord blood sample) in future siblings. 

• Providing the family with visual evidence that their baby (who is often asymptomatic) has a lifelong disorder is helpful.

• Giving a firm diagnosis (achievable in 80% of our cases by initial scanning) not only resolves uncertainty and removes the need for further investigation in later childhood, but also reinforces the need for full compliance with lifelong medication.

• Determining the precise diagnosis may inform subsequent T4 dosing.

The potential pitfalls of both thyroid ultrasound and radioisotope scanning must be recognised. We and others have drawn attention to the diagnostic trap of misdiagnosing non-thyroidal tissue in thyroid fossa as thyroid dyplasia/hypoplasia (51,52). However, this problem can easily be avoided by recognising the hyperechoic nature of the non-thyroidal tissue (similar to that of subcutaneous fat), heterogeneous texture of the echo signal, poor vascularity, presence of anechoic and/or hypoechoic cysts, and extension of the tissue both around and behind the large cervical blood vessels (51). The commonest pitfall of isotope scanning is lack of uptake despite the presence of thyroid tissue, leading to the spurious diagnosis of athyreosis. This phenomenon is seen when the scan is delayed beyond 4-5 days of T4 treatment so that TSH suppression has occurred by the time the scan is performed; from iodine exposure, for example, the application of topical iodine for antiseptic procedures; from blocking TSH-receptor antibodies causing transient hypothyroidism; and (rarely) from defects affecting iodine uptake, e.g. sodium-iodide symporter gene mutations. The diagnosis of spurious thyroid absence will not be made if concurrent ultrasound examination is carried out. Also, venous TSH should always be measured at the time of thyroid imaging, so that the isotope uptake can be correctly interpreted. 

**Further Investigation**

If thyroid imaging shows an ectopic thyroid gland, or athyreosis with unrecordable TG, then further investigation is not usually required. The finding of a normal or hypoplastic gland in situ with either reduced or absent isotope uptake raises the possibility of allo-immune thyroid dysfunction, and further blood from both mother and child should be taken for thyroid peroxidase and TSH-receptor antibody testing. Transplacental passage of maternal antibodies may cause transient CH accounting for 2.7% of referrals in one series, some of whom were siblings (53) and, very rarely, permanent hypothyroidism. A small in situ thyroid gland should also prompt DNA analysis for a TSH-receptor defect, and there is also a case for this analysis in apparent athyreosis with normal or high TG levels. 

A normal or large gland in situ, especially if radioisotope uptake is increased, suggests dyshormonogenesis due to an inherited enzyme defect. In this situation, blood from parents and infant should be taken for DNA extraction and mutational analysis (see algorithm). However, the pattern of large/normal gland in situ with avid isotope uptake is also seen in iodine deficiency. At present in the UK, the iodine status of infant and mother is not routinely assessed, but data from the North East of England indicate iodine deficiency in 3.5% of pregnant women with borderline deficiency in 40% (54). More recent work in Scotland from Bannon and Aitken has shown urinary iodide levels below the WHO cut-off of 150 μg/L in 65% of 1097 pregnant women recruited between 2008-2010 (Bannon, 2012 in preparation). We are planning a prospective study in which maternal and infant urine and serum iodine will be measured in all Scottish infants referred with TSH elevation, to exclude the possibility that some cases of mild TSH elevation with normal or large thyroid gland in situ are related to iodine insufficiency rather than mild dyshormonogenesis. 

**Further Counselling**

The second visit, 4-5 days after initial assessment, is an opportune time to give the parents a more detailed verbal account of CH and to let them see the diagnostic images of their baby in order to reinforce the reality of the condition. We also give out a booklet, freely available from the Child Growth Foundation website (www. childgrowthfoundation.org/cgf_ booklets - Thyroid disorders) and obtain written informed consent for their child’s data to be entered onto our database. 

**Subsequent Management and Follow-up (After InitialDiagnosis and Treatment)**

**Initial Dose Titration**

Following the initial and second visit, we advise weekly assessment including thyroid function tests until the venous TSH has fallen to below 5 mU/L, aiming to normalise TSH within 14 days, as recommended by Selva et al (55). During these first few weeks, we allow the fT4 level to be above the reference range, but thereafter, the T4 dose is titrated to keep free T4 between 15 and 26 pmol/L and TSH between 0.5 and 5 mU/L. These limits ensure adequate treatment but avoid overtreatment, with the risk of an adverse effect on attention and behaviour in later childhood (18). The T4 dose will usually need to be reduced to 37.5 μg daily or less following an initial period (5-10 days) of high-dose therapy to achieve rapid TSH reduction. 

**Subsequent Dose Scheduling**

Once the TSH has normalised, we recommend calculating surface area using the formula ? [length (cm) x weight (kg)/3600] and tailoring the replacement dose to the child’s needs, which will depend on the type and severity of CH. We consider it more logical to increase the dose pre-emptively rather than allowing the brain to ‘see’ too little T4, thus provoking a rise in TSH and prompting an increase in dosage. 

The full replacement dose (e.g. in athyreosis) is around 100 μg/m2/day (3-4 μg/kg/day), but infants with milder disease (e.g. lingual ectopia with compensated hypothyroidism pre-treatment) will require less. A suggested dosage schedule, based on the requirement in the absence of thyroid tissue, is given in Table 3. 

Most children do well when free T4 is kept between 9-26 pmol/L and TSH to between 0.5 and 5 mU/L, but some seem to be more sensitive to T4. Thus, parents need to be aware of the symptoms of T4 overtreatment, which include poor sleeping, heat intolerance, and irritability. 

**Frequency and Structure of Clinic Visits**

Our suggested schedule for each visit is outlined in Table 4. Ideally, the child should undergo venipuncture a week or so before the clinic visit so that the results of thyroid function tests are available at the consultation, but not all families are able or willing to schedule two visits. The child’s physical growth and neurodevelopment are assessed by auxology and a developmental/educational enquiry. School performance can be gauged by enquiring as whether or not the pupil is in an age-appropriate year, or if he/she has been held back; overall educational status compared with peers; areas of vulnerability including mathematics and reading; and the need of extra support. The clinic visit also provides an opportunity to counsel and educate the parents, and subsequently the child/adolescent. 

We do not routinely carry out pubertal assessment or bone age estimation in our patients since we would not normally do this assessment either in normal children and adolescents, and the patients with CH can be regarded as normal individuals who simply need to be on replacement treatment. Given the concerning reports of hearing impairment in adults with CH (32), we are considering referral at 5 years for formal audiology in all patients with severe or moderate CH. 

**Re-evaluation of Patients with TSH Elevation of Uncertain Cause**

Patients with clear evidence of thyroid dysgenesis on diagnostic imaging, or episode(s) of frank TSH elevation (> 15 mU/L) beyond infancy despite T4 treatment, do not normally require retesting. Children who do require re-evaluation can be broadly grouped into three categories; those who: 

• Were preterm and/or sick at the time of testing 

• Have congenital malformations and/or dysmorphic syndromes (including Down’s syndrome)

• Show mild TSH elevation of uncertain cause +/- normal gland in situ

Re-evaluation should be deferred until 2-3 years of age, depending on the severity of TSH elevation at initial assessment. Treatment can be reduced and stopped over a 4- week period and thyroid function tested 2 weeks later. Diagnostic imaging can be carried out at this stage if no previous imaging has been performed. 

**Transition to Adult Care**

The age at transition from paediatric to adult care is at the discretion of the clinician and family. It is important for all patients to understand their condition and the long-term health implications of good compliance with T4 treatment. Girls with CH also need to be aware of the importance of preconceptual euthyroidism so that the foetus receives sufficient T4 during the first trimester. We therefore arrange for girls to be seen by an adult endocrinologist in our transition clinic, whereas we would normally aim to discharge boys to the care of their general practitioner at this age. 

**Conclusions**

The outlook in primary CH is generally good, and it is likely that the advent of earlier and higher dose treatment over the past two decades will be shown to have improved outcome. The assessment of both the impact of disease severity and the degree to which this can be offset by postnatal management is difficult and requires long-term follow-up into adulthood, by which time practice is bound to have changed. Although much can be learned from the admirable French cohort studies of Léger et al (32,34), there is a dearth of large-scale, prospective studies in which children have been systematically treated according to agreed protocols. There is an urgent need in Europe for new consensus guidelines to update previous ESPE recommendations (49). Thereafter, children treated according to such guidelines and carefully stratified according to disease severity need systematic follow-up, with assessment at agreed time points, so that good quality data on outcome can be collected. 

**Acknowledgements**

We wish to thank Dr Tim Cheetham, Mrs Joan Mackenzie, Emma Jane Gault, and Denise Bannon for their help in preparing this review. We also thank our paediatric colleagues from all over Scotland for their support and input into our congenital hypothyroid database. 

## Figures and Tables

**Table 1:**
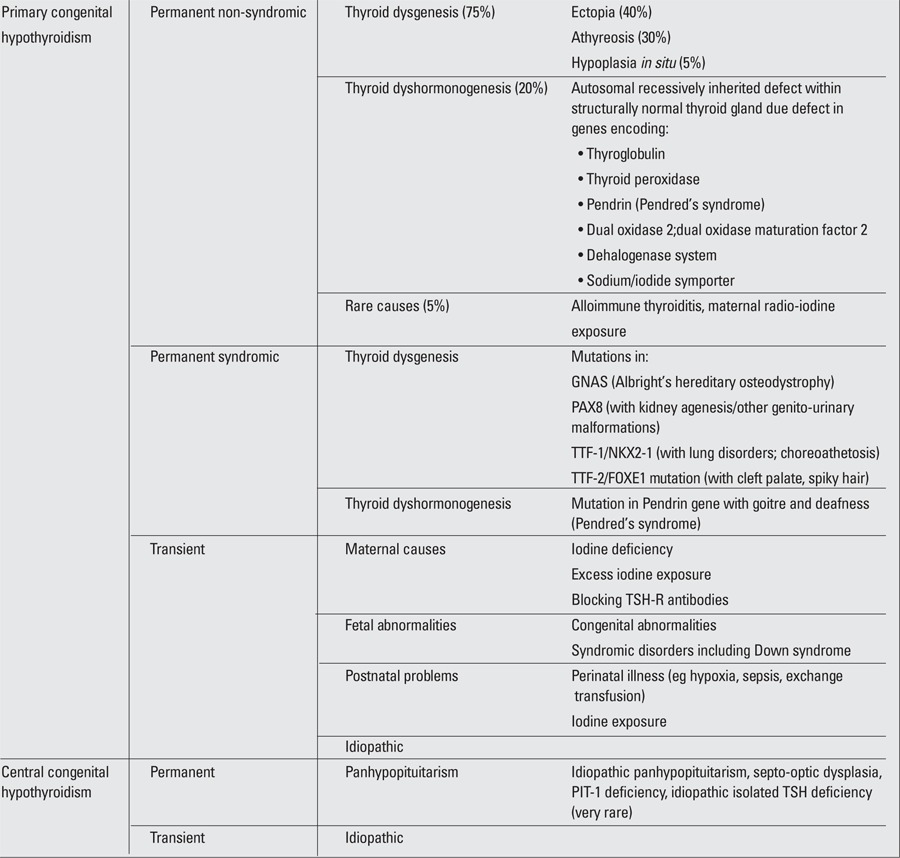
Causes of congenital hypothyroidism, classified according to site (primary or central), type (non-syndromic and syndromic) and duration(permanent and transient)

**Table 2:**
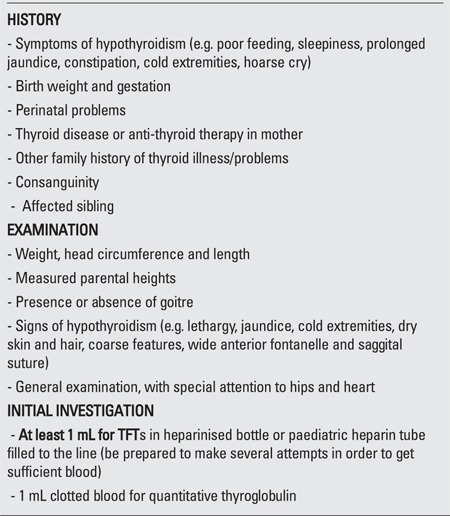
Initial clinical assessment of infant referred with capillary thyroidstimulating hormone (TSH) elevation

**Table 3:**
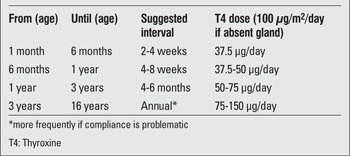
Suggested follow-up and dose schedule from 1 month of age toadult transfer

**Table 4:**
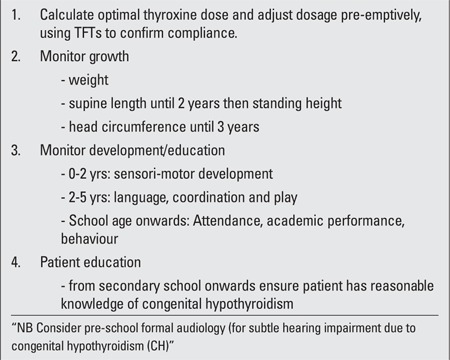
Recommended schedule for each outpatient visit in children withcongenital hypothyroidism
